# Fucoidans Disrupt Adherence of *Helicobacter pylori* to AGS Cells *In Vitro*

**DOI:** 10.1155/2015/120981

**Published:** 2015-10-28

**Authors:** Eng-Guan Chua, Phebe Verbrugghe, Timothy T. Perkins, Chin-Yen Tay

**Affiliations:** The Marshall Centre for Infectious Diseases Research and Training, School of Pathology and Laboratory Medicine (M502), The University of Western Australia, 35 Stirling Highway, Nedlands, WA 6009, Australia

## Abstract

Fucoidans are complex sulphated polysaccharides derived from abundant and edible marine algae. *Helicobacter pylori* is a stomach pathogen that persists in the hostile milieu of the human stomach unless treated with antibiotics. This study aims to provide preliminary data to determine, *in vitro*, if fucoidans can inhibit the growth of *H. pylori* and its ability to adhere to gastric epithelial cells (AGS). We analysed the activity of three different fucoidan preparations (*Fucus* A, *Fucus* B, and *Undaria* extracts). Bacterial growth was not arrested or inhibited by the fucoidan preparations supplemented into culture media. All fucoidans, when supplemented into tissue culture media at 1000 *µ*g mL^−1^, were toxic to AGS cells and reduced the viable cell count significantly. Fucoidan preparations at 100 *µ*g mL^−1^ were shown to significantly reduce the number of adherent *H. pylori*. These *in vitro* findings provide the basis for further studies on the clinical use of sulphated polysaccharides as complementary therapeutic agents.

## 1. Introduction

Fucoidan, derived from marine edible brown algae, is a complex sulphated polysaccharide [1]. The structures and compositions of fucoidan vary across different brown algae species, but they consist primarily of L-fucose and sulphate, which form polymers with small quantities of D-galactose, D-mannose, D-xylose, and uronic acid [1, 2]. Studies on fucoidans' propensity to alter biological processes associated with disease have included analysing their ability to inhibit tumour growth, modulate the immune system, interfere with viral mechanisms, and inhibit coagulation and their use as a reducing agent or antioxidant [2–10].

Many algae-derived concoctions used in traditional medicine have been recorded in pharmacopoeias as agents used to treat bacterial infection, either by inhibiting growth or by colonisation. Indeed, the antibiotic nature of fucoidan has been scientifically investigated* in vitro* for its ability to inhibit the growth of bacteria that are commonly known to develop multidrug resistance such as* Staphylococcus aureus* and* Escherichia coli* [11–13]. Various sulphated polysaccharides including heparin, heparin oligosaccharides, and fucoidan were also reported to competitively inhibit the colonisation of the gastric pathogen* Helicobacter pylori* (*H. pylori*) [14, 15]. Furthermore, the adherence of* Helicobacter* species to macrophages was inhibited by fucoidan [16].

*H. pylori* is a Gram-negative bacterium that colonises the stomach of half of the world's human population. It causes chronic active gastritis which can progress to peptic ulcers, gastric cancer, and gastric MALT lymphoma [17]. The host's immune system is unable to clear the infection and it persists unless treated. Current standard* H. pylori *infection therapy consists of administration of a proton pump inhibitor and two antibiotics, amoxicillin and clarithromycin or metronidazole [18]. It was, however, recently shown that the efficacy of this empiric triple therapy has declined to an unacceptably low level of 70% compared to at least 90% eradication rate as a general rule for therapeutic regime prescription against infectious diseases [19]. This problem is in fact due to the increased prevalence of clarithromycin resistance in* H. pylori *isolates worldwide and it is expected that such occurrence would continue to surge with the use of eradication therapy [20–22]. Treatment regimens with higher success rates can involve using expensive and often restricted antibiotics [23]. Alternative and complementary strategies may improve the success rate of current treatment regimens by providing an alternative mechanism to reduce the bacterial burden.

In this study, we investigated the antimicrobial activity of three fucoidan extracts, two of which were isolated from* Fucus vesiculosus *and one from* Undaria pinnatifida*, against* H. pylori* and how these fucoidans can alter adherence of* H. pylori* to human gastric epithelial cells* in vitro*.

## 2. Materials and Methods

### 2.1. Bacterial Culture Conditions

*Helicobacter pylori* strain NCTC 11637 was routinely cultured on Columbia agar supplemented with 7% (v/v) defibrinated horse blood (PathWest) at 37°C in a 10% CO_2_ environment.

### 2.2. Mammalian Cell Culture

Human gastric adenocarcinoma epithelial cells (AGS) (Sigma) were cultured in RPMI-1640 medium (Gibco, Invitrogen) supplemented with 20 mM L-glutamine and 10% (v/v) heat-inactivated fetal bovine serum (FBS) (Serana, Fisher Biotech).

### 2.3. Biochemical Composition Analysis

Three different fucoidans used in this study were kindly provided to us by Marinova Pty. Ltd. (Australia), two of which were derived from* Fucus vesiculosus *and one from* Undaria pinnatifida*.

Total carbohydrate content was determined by spectrophotometric analysis of the hydrolysed compound in the presence of phenol, based on a method described by Dubois et al. [24].

Sulfate content was analysed spectrophotometrically using a BaSO_4_ precipitation method (BaCl_2_ in gelatin), based on the work of Dodgson [25, 26]. In brief, samples were heated in 1 M hydrochloric acid solution at 105–110°C for 3 hours to adequately cleave all sulfate groups from the molecule. These were then precipitated using a BaCl_2_/gelatin mixture, and concentration was determined by UV-Vis.

Analysis of polyphenol content was also determined spectrophotometrically, using a method based on the Folin-Ciocalteu reagent (phosphomolybdate/phosphotungstate) [27–29].

The monosaccharide composition was determined using a GC-based method for the accurate determination of individual monosaccharide ratios in a sample. This method relies on the preparation of acetylated alditol derivatives of the hydrolysed samples [30].

Molecular weight profiles were determined by Gel Permeation Chromatography, with the aid of a Size-Exclusion Column, and were reported relative to Dextran standards.

### 2.4. Bacterial Growth Inhibition Assay

*H. pylori *NCTC 11637 bacteria were inoculated (OD_600_ = 0.05) in duplicate, into 5 mL of Brain Heart Infusion Broth (BHIB) (Oxoid) containing 10% (v/v) heat-inactivated FBS,* H. pylori* selective supplement (DENT) (Oxoid), and individual fucoidan in final concentrations of 1, 10, 100, and 1000 *μ*g mL^−1^. These cultures were prepared in T25 cell culture flasks (Nunc) and incubated in microaerophilic condition with moderate agitation at 37°C. Aliquots were taken over time and the OD_600_ readings were measured.

### 2.5. Cell Toxicity Assay and Viable Cell Count

Cells were seeded at 5 × 10^4^ cells per well in a 24-well tissue culture plate (Corning CellBIND Surface) and incubated at 37°C in a 5% CO_2_ atmosphere until confluent monolayers formed. Three different fucoidans, each prepared in RPMI medium at the concentrations 1, 10, 100, and 1000 *μ*g mL^−1^, were added to the cell monolayers in triplicate to test for cell cytotoxicity. Cells were incubated for 3 hours and the cell viability was determined by measuring lactate dehydrogenase (LDH) release using the CytoTox 96 nonradioactive cytotoxicity assay (Promega) and by using the trypan blue (Sigma) exclusion method, a stain that selectively colours viable cells.

### 2.6. *Helicobacter pylori* Adherence Assay

AGS cells were grown to confluence in 24-well plates.* H. pylori* cells (grown 24 hours on blood agar plates at 10% CO_2_ and 37°C) were harvested and resuspended in RPMI medium to form the inoculum. The monolayers were inoculated with bacterial suspension at a multiplicity of infection (MOI) of 10 : 1. 3 hours after incubation; the cells were washed and incubated with fucoidans of 100 *μ*g mL^−1^, in triplicate, for 3 hours under the same conditions. Cells were washed 3 times with phosphate-buffered saline (PBS) (Gibco, Invitrogen) and lysed with 1% (w/v) saponin (Sigma) in PBS for 15 minutes. Dilutions of cell lysates were plated on blood agar plates and incubated at 37°C in a 10% CO_2_ environment for 2-3 days. Visible bacterial colonies were counted. The experiment was repeated twice on different occasions. Results were presented as the mean of three assays with standard deviation.

### 2.7. Statistical Analysis

Statistical analysis was carried out using Student's *t*-test (Microsoft Excel software). *P* values of <0.05 were considered statistically significant.

## 3. Results and Discussion

### 3.1. Biochemical Content Analysis

Fucoidan, derived primarily from marine brown algae, is a complex sulphated polysaccharide and it has been extensively studied over the past three decades for its wide range of potentially beneficial biological properties. Depending on the source of algae, there is considerable diversity in the basic composition and structure of fucoidans and such structural composition variation confers different or unique biological functions to these diverse sulphated compounds [31].

Three fucoidan extracts, two of which were fucoidan fractions isolated from* Fucus vesiculosus* and one from* Undaria pinnatifida*, were examined for their total contents including carbohydrate, sulfate, and polyphenol. These data were supplied to us by Marinova and the relative percentages are described in Table 1. Biochemical composition analysis demonstrated that both* Fucus* A and* Fucus *B are highly sulfated fucose polymers.* Fucus *A contained mainly fucose (59.4%) and sulphate (25.3%) and much smaller amounts of galactose and polyphenol (3.3% and approximately 3-4%, resp.).* Fucus *B, however, contained nearly 50% less fucose level (31%) and around 8-fold greater polyphenolic antioxidant compositions than in* Fucus *A. The* Undaria *fucoidan was shown to be composed of mainly fucose, galactose, and sulphate with 42.4%, 22.5%, and 26.3%, respectively, as well as a small amount of polyphenol (2.5%).

### 3.2. Fucoidans Do Not Inhibit the Growth of* H. pylori* in Culture

Fucoidan derived from* Laminaria japonica* was reported to be bacteriostatic against several microorganisms including* S. aureus*. Furthermore, in a study conducted by Lee et al. [32], the combination of antibiotics and fucoidan demonstrated synergistic effect on oral pathogenic bacterial killing. In light of these studies, the potential of* Fucus *A,* Fucus *B, and* Undaria *extracts was investigated in the context of* H. pylori* eradication.

**Figure 1:**
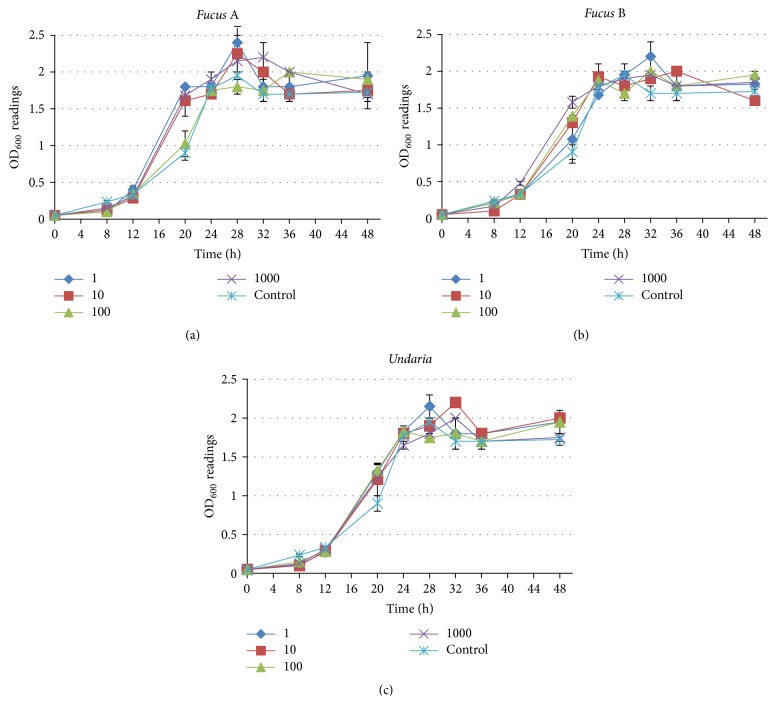
Growth curve analysis of* H. pylori* NCTC 11637 cultured under varying concentrations (ranging from 1 to 1000 *μ*g mL^−1^) of different fucoidan extracts. The *y*-axis represents the OD_600_ reading over time (*x*-axis, hours). (a)* H. pylori *NCTC 11637 grown in the presence of* Fucus *A extract. (b)* H. pylori *NCTC 11637 grown in the presence of* Fucus *B extract. (c)* H. pylori *NCTC 11637 grown in the presence of* Undaria* extract. None of the fucoidan preparations displayed antibacterial growth activity on* H. pylori *NCTC 11637.

To determine if fucoidan extracts are bacteriostatic or bactericidal,* H. pylori* strain NCTC 11637 was cultured in growth medium supplemented with* Fucus *A,* Fucus *B, or* Undaria *extract at different concentrations up to 1000 *μ*g mL^−1^. The growth of* H. pylori *did not differ in the presence of fucoidans compared with the control (growth medium only) (Figure 1), indicating that no bacteriostatic or bactericidal activity was observed for any of the fucoidan preparations against* H. pylori*.

### 3.3. Fucoidans Are Cytotoxic to AGS Cancer Cells at Higher Concentrations

Since inhibition of bacterial growth was not observed, we then considered whether the fucoidans are capable of inhibiting* H. pylori* adherence* in vitro* to a commonly used gastric epithelial cell line, AGS cells. Bacterial attachment to host cells is a key process in bacterial pathogenesis or infection and inhibiting this interaction would reduce bacterial load significantly as* H. pylori *is incapable of colonising alternative niches in the body. Initially, we investigated the cytotoxicity of fucoidans on AGS cells, as it has been shown in numerous studies that fucoidans inhibit carcinoma cell proliferation [33–40]. We, therefore, identified the safe range of fucoidan concentrations used in this assay to ensure maximal cell viability and thus consistency in the number of adherent bacteria recovered after sampling with fucoidans.

**Figure 2:**
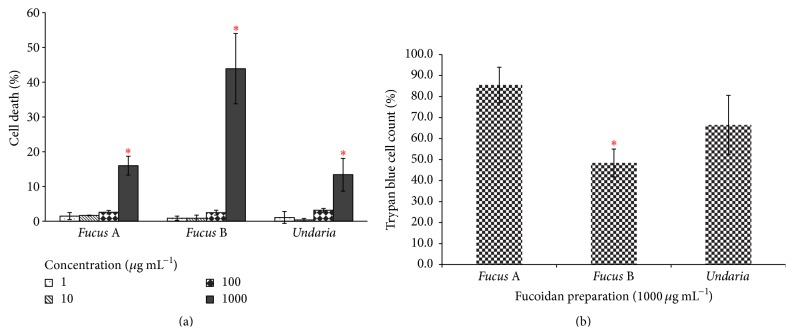
Cytotoxicity of fucoidans. (a) Lactate dehydrogenase (LDH) cytotoxicity assay on AGS cells. Error bars represent standard deviation whilst the symbol *∗* indicates statistical significance when compared to the untreated sample (*P* value < 0.05). (b) Trypan blue viability test. Viable AGS cells after being treated with fucoidan preparations of 1000 *μ*g mL^−1^were counted using trypan blue exclusion method. Untreated AGS cells were defined as 100% relative growth.

To determine if the fucoidan preparations induce cell death, their cytotoxicity of AGS cells was tested by measuring the presence of lactate dehydrogenase (LDH) in the supernatants after exposure (Figure 2(a)) [41]. The levels of LDH were significantly higher for all fucoidan extracts at the concentration of 1000 *μ*g mL^−1^. Viability counting using trypan blue exclusion approach indicated that 85.5% of cells were viable when* Fucus *A extract was added to the AGS cells and that 66.5% were viable in the presence of the* Undaria *preparation (Figure 2(b)). Fewer viable cells were counted for the* Fucus *B extract (48.4%) than for the* Fucus* A and* Undaria* extracts.

All 3 fucoidan preparations were found to be toxic to AGS cells when tested at the concentration of 1000 *μ*g mL^−1^. This is in line with previous studies that fucoidan induces cell death in a dose-dependent manner in several carcinoma cell lines, including AGS cells. Changes in apoptotic regulation are thought to be the mechanism behind this fucoidan-mediated cell death [36, 38, 40, 42]. It is also important to mention that* Fucus *B extract exerted the greatest cell cytotoxicity towards AGS cells, which is likely due to its high polyphenol content that is at least 8-fold greater compared to* Fucus *A and* Undaria*. Polyphenol antioxidant has been extensively reported for its anticancer properties and such activity is thought to be mediated through inhibition of kinase activity [43].

### 3.4. Fucoidans Disrupt* H. pylori* Adherence to AGS Cells

**Figure 3:**
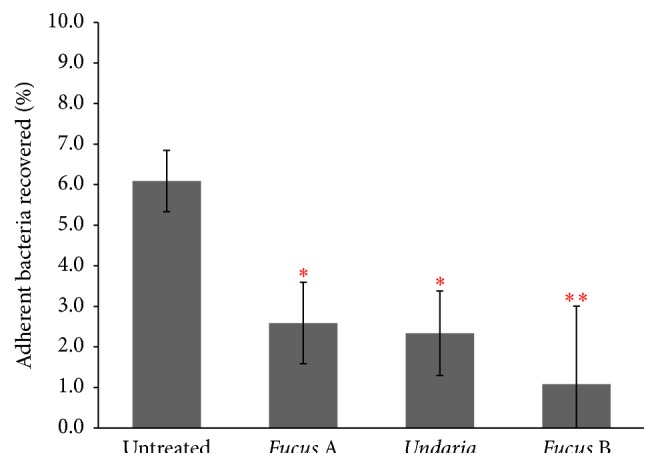
Treatment of AGS cells with each fucoidan at 100 *μ*g mL^−1^ following* H. pylori *inoculation. The symbols *∗* and *∗∗* represent statistical significance of *P* values less than 0.05 and 0.01, respectively, with respect to untreated control.

We inoculated AGS cells with* H. pylori *allowing them to bind to the cells and then washed the confluent cells to maintain the* H. pylori* that were adhered to the AGS cells. Using this aggregate, we supplemented the replacement growth media with each type of fucoidan at 100 *μ*g mL^−1^, incubated the cells, and subsequently washed the cells to remove nonadherent bacteria. The numbers of colony forming units (CFUs) were counted compared to the control's CFUs for each experiment to determine if the fucoidan disrupted the adhered bacteria. All fucoidan extracts were shown to significantly remove adherent bacteria from the AGS cell surface when tested at 100 *μ*g mL^−1^ (*P* < 0.05 for* Fucus *A and* Undaria* and *P* < 0.01 for* Fucus *B) (Figure 3). This finding indicates that fucoidans bind either to* H. pylori *or to AGS cells with stronger affinity, thus putatively dislodging the bacteria from host cell surface. It is, however, strongly believed to be the former event as it has been reported that fucoidan extracted from* Cladosiphon okamuranus *TOKIDA brown algae, which similarly inhibited* H. pylori *binding to human gastric cell lines MKN28 and KATO III, exerted more potent inhibitory effect by preincubation with bacteria but failed to reduce bacterial adhesion by pretreatment of gastric cells [44]. Furthermore, in the same study, several fucoidan-binding* H. pylori* outer membrane proteins were detected by immunoblotting analysis. In another study which investigated the effect of* Fucus vesiculosus *derived fucoidan and sulphated polysaccharides including heparin and Dextran on enterohepatic* Helicobacter *species to murine macrophage cell line J774A.1, it was shown that only fucoidan achieved substantial reduction of bacterial binding to host cells [16]. The results above provide further support that the anti-*H. pylori *adhesion activity demonstrated in our study is fucoidan-specific, not due to an unspecific colloidal effect.

## 4. Conclusions

Taken together, the results show that fucoidans inhibit* H. pylori *attachment to gastric epithelial cells* in vitro*. Furthermore, we demonstrated that* Fucus *B, a secondary fucoidan fraction with high polyphenol content extracted from* Fucus vesiculosus*, is the most toxic against AGS carcinoma cells. Hence, if fucoidan, especially* Fucus *B, can penetrate the protective mucosal surface of the stomach to bind to* H. pylori *at a low pH, it has potential clinical applications in the treatment of* H. pylori *infection, as well as potential to prevent gastric cancer development. More importantly, other fucoidan preparations appear to be nontoxic with daily intake up to 6 grams in human clinical studies [45]. Future studies in* in vivo* infection models are necessary to assess whether the use of these fucoidans in addition to antibiotics would improve* H. pylori *eradication efficiency.

## Figures and Tables

**Table 1:** Biochemical composition of fucoidan preparations. The columns detail the proportions of fucose, galactose, sulphate, and polyphenol that are present in the fucoidan component of the sample.

Extract	% sulfate	% fucose	% galactose	% polyphenol	Peak MW (kDa)
*Fucus* A	25.3	59.4	3.3	3-4	61.5
*Fucus* B	21.8	31.0	2.6	26.2	203.6
*Undaria*	26.3	42.4	22.5	2.5	100.9
